# Impact of Plasma 5 Hydroxyindoleacetic Acid, a Serotonin Metabolite, on Clinical Severity in Acute Respiratory Distress Syndrome

**DOI:** 10.3389/fmed.2021.785409

**Published:** 2021-12-16

**Authors:** Takeshi Tanaka, Masahiko Mori, Masato Tashiro, Koichi Izumikawa

**Affiliations:** ^1^Infection Control and Education Center, Nagasaki University Hospital, Nagasaki, Japan; ^2^Department of Paediatrics, University of Oxford, Oxford, United Kingdom; ^3^Department of Infectious Diseases, Nagasaki University Graduate School of Biomedical Sciences, Nagasaki, Japan

**Keywords:** ARDS, serotonin, 5-HIAA (5-hydroxyindoleacetic acid), vascular permeability, shock

## Abstract

Acute respiratory distress syndrome (ARDS) is characterized by dysregulated vascular permeability. The clinical outcomes remain poor, and the disease burden is widespread. We demonstrated that plasma 5-hydroxyindoleacetic acid (5-HIAA), a serotonin metabolite, is a pivotal severity indicator of ARDS. Serotonin is an effector of cellular contraction and a modulator of vascular permeability. Plasma 5-HIAA levels were significantly elevated in severe ARDS cases with shock status (*p* = 0.047) and positively correlated with SOFA (*p* < 0.0001) and APACHE-II score (*p* < 0.0001). In the longitudinal analysis, plasma 5-HIAA levels were also a strong independent predictor of mortality rate (*p* = 0.005). This study indicates that plasma 5-HIAA is a biomarker of ARDS severity and highlights the importance of evaluating vascular leakage levels for ARDS treatment.

## Introduction

The concept of acute respiratory distress syndrome (ARDS) was first introduced over 50 years ago (1); however, its mechanism of pathogenesis remains poorly understood, while its disease burden is substantial. In addition, a large number of cases are complicated by septic shock, which further increases morbidity and mortality. ARDS and septic shock are characterized by dysregulated vascular permeability (2). One of the major mechanisms that regulate vascular permeability is cellular contraction (3), which can be induced via the serotonin and RhoA/Rho-associated protein kinase (ROCK) signaling pathway in certain cell types (4). Serotonin (5-hydroxytryptamine [5-HT]) is a classical neurotransmitter in the central nervous system; however, 95% of 5-HT production in the body is generated in peripheral tissues, where a variety of pleiotropic effects are elicited, including vasoconstriction, proliferation, and inflammation (5).

We recently reported the effect of plasma 5-hydroxyindoleacetic acid (5-HIAA), a serotonin metabolite, on the clinical outcomes of sepsis (6), with an increase in plasma 5-HIAA levels in patients with severe septic shock. In addition, we demonstrated the potential role of serotonin in vascular permeability through *in vitro* ROCK activation experiments (6). Burdens of vascular leak remain a large issue in several diseases, such as ARDS (any etiology including COVID-19), viral hemorrhagic fever, and dengue fever. Our conceptual hypothesis of the serotonin-ROCK pathway approach to regulating vascular permeability could be a common approach for all types of these diseases. In this study, plasma 5-HIAA measurements were performed using high-performance liquid chromatography on plasma samples from 157 ARDS patients. In addition, we statistically analyzed its association with disease severity and mortality.

## Methods

Plasma samples were collected on day 0 from 157 randomly selected ARDS patients, and the SAILS Research Materials dataset was obtained from the National Heart, Lung, and Blood Institute (NHLBI) Biologic Specimen and Data Repository Information Coordinating Center, USA. Data sets that were analyzed in this report were from patients who were diagnosed with ARDS on day 0, had a ratio of the partial pressure of arterial oxygen (PaO_2_) to the fraction of inspired oxygen (FiO_2_) of 300 or less, and had bilateral infiltrates on chest radiography that were consistent with pulmonary edema without evidence of left atrial hypertension (7). Plasma 5-HIAA levels were measured by high-performance liquid chromatography at the SRL laboratory (Tokyo, Japan). Baseline information and disease severity indices of the Sequential Organ Failure Assessment (SOFA) score from 137 subjects, and Acute Physiology and Chronic Health Evaluation (APACHE) II score from 157 subjects were obtained from the NHLBI dataset. Statistical analysis was performed using GraphPad Prism, version 6.07 (San Diego, CA, USA). Between alive and death groups during 90 days of follow-up, Fisher's exact tests were used for sex and shock distribution difference analyses, and Mann–Whitney *U*-tests were used for age, SOFA score, APACHE II score, and plasma 5-HIAA level difference analyses. Analysis of plasma 5-HIAA level differences between ARDS patients with and without shock was performed using the Mann–Whitney *U*-test. Spearman's correlation test was used to analyze the correlations between disease severity indices (SOFA score and APACHE II score) and plasma 5-HIAA levels. Further, for the survival rate analyses, we constructed a binary logistic regression model for cross-sectional analysis and a Cox hazard model for longitudinal analysis.

## Results

**Figure 1:**
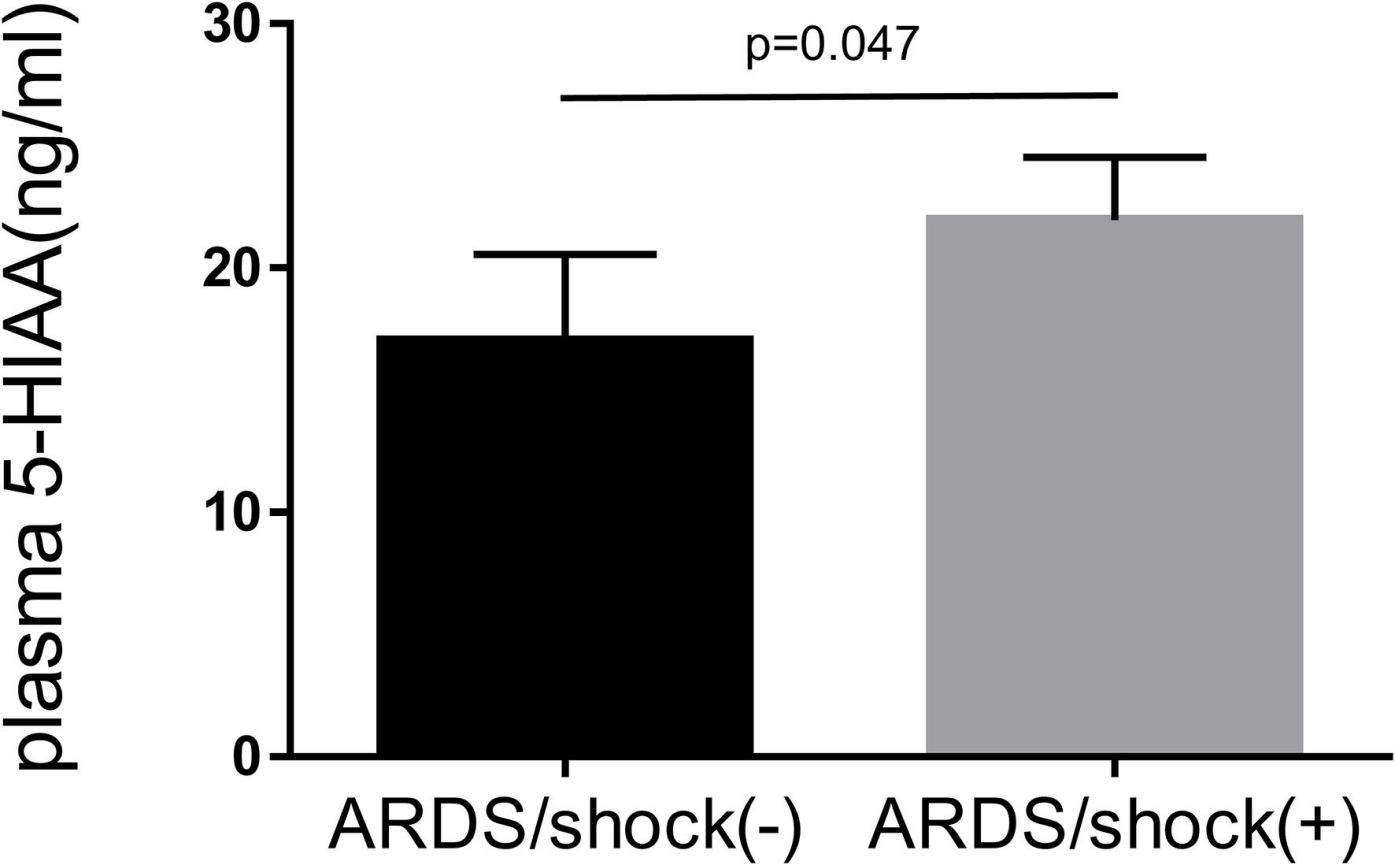
Plasma 5-HIAA levels in patients with ARDS. Comparison of plasma 5-HIAA levels between ARDS/shock+ and ARDS/shock- patients was performed using the Mann–Whitney *U*-test the 5-HIAA levels were higher in the ARDS/shock+ group compared to the ARDS/shock- group (median 7.1 ng/mL vs. 11.5 ng/mL, *p* = 0.047).

**Figure 2:**
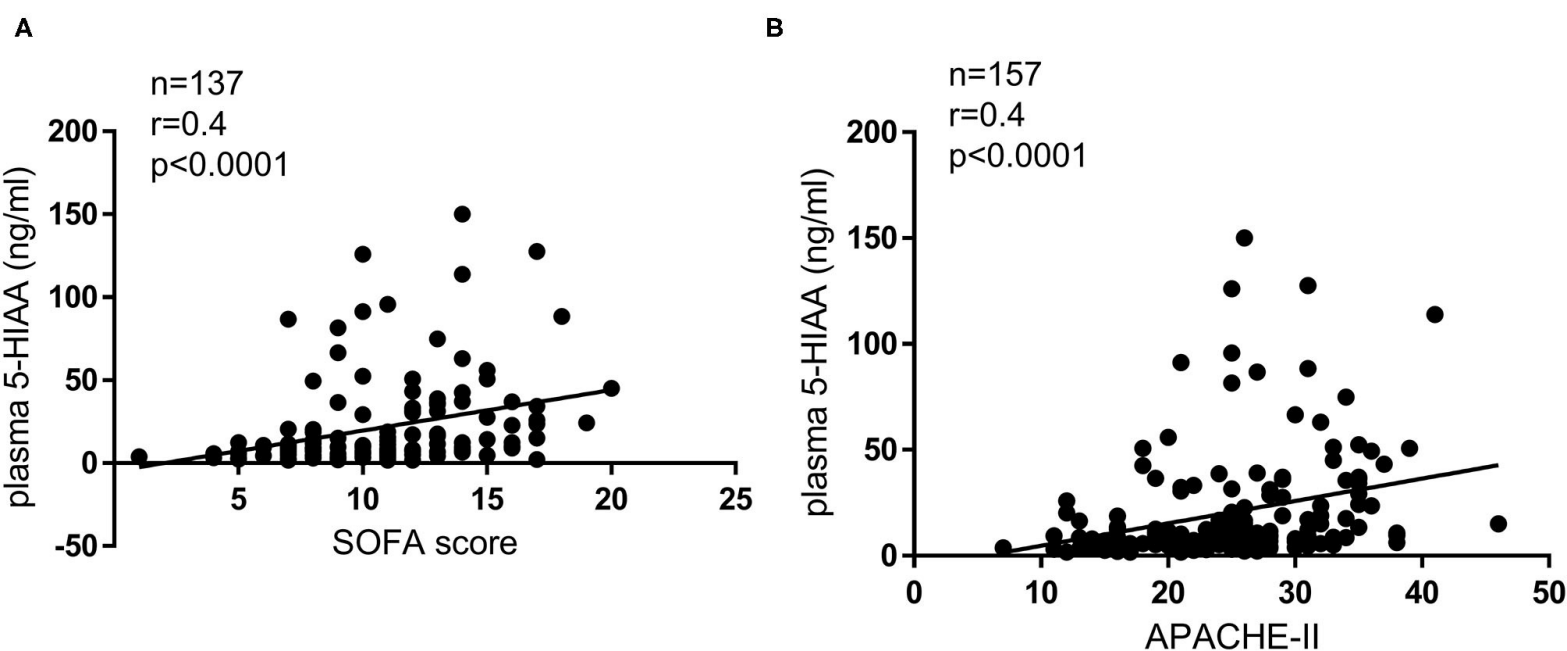
Correlation between disease severity indices and plasma 5-HIAA levels. Correlation between SOFA score and plasma 5-HIAA level **(A)**, APACHE II score, and plasma 5-HIAA level **(B)**; Spearman's correlation test. A positive correlation between plasma 5-HIAA levels and clinical disease severity indices was observed (*r* = 0.40, *p* < 0.0001, *r* = 0.40, *p* < 0.0001).

Baseline clinical characteristics are shown in Table 1. Between alive and death groups, significant differences were identified in age (median 55 years old in alive group vs. median 63 years old in death group, *p* = 0.004), shock status (61 vs. 87%, *p* < 0.001), APACHE-II score (24 vs. 26, *p* = 0.004), and plasma 5-HIAA level (8.6 ng/ml vs. 14.6 ng/ml, *p* < 0.001). The plasma 5-HIAA levels were higher in the ARDS/shock+ group compared to the ARDS/shock- group (11.5 ng/mL vs. 7.1 ng/mL, *p* = 0.047) (Figure 1). Plasma 5-HIAA levels were positively correlated with SOFA scores (*r* = 0.40, *p* < 0.0001) (Figure 2A) and APACHE II scores (*r* = 0.40, *p* < 0.0001; Figure 2B). In the cross-sectional survival rate analysis, age (odds ratio [OR] 1.03, *p* = 0.005), shock status (OR 4.1, *p* = 0.002), APACHE-II score (OR 1.1, *p* = 0.005), and plasma 5-HIAA level (OR 1.03, *p* < 0.001) were significantly associated with mortality (Table 2), while shock (OR 3.7, *p* = 0.008) and plasma 5-HIAA levels (OR 1.02, *p* = 0.005) remained significant in the multivariate analysis (Table 2). In the longitudinal analysis, age (hazard ratio [HR] 1.03, *p* = 0.004), shock status (HR 3.3, *p* = 0.003), APACHE-II score (HR 1.06, *p* = 0.004), and plasma 5-HIAA level (HR 1.01, *p* < 0.001) were significantly associated with mortality in the univariate analyses (Table 3). In multivariate analysis, plasma 5-HIAA level (adjusted hazard ratio [aHR] 1.01, *p* = 0.005) as well as shock status (aHR 2.7, *p* = 0.02) remained significantly associated with mortality (Table 3). These results strongly suggest that plasma 5-HIAA levels could be an indicator of disease severity in patients with ARDS.

**Table 1:** Characteristics of 157 ARDS patients.

**Characteristics**	**All (*n* = 157)**	**Alive (*n* = 105)**	**Death (*n =* 52)**	***p***
Sex (female)	82 (52%)	56 (53%)	26 (50%)	0.7
Age	^a^59 (46–68)	^a^55 (43–65)	^a^63 (55–71)	0.004
Shock	109 (69%)	64 (61%)	45 (87%)	<0.001
SOFA score (*n =* 137)	^a^11 (8–13)	^a^11 (8–13) (*n =* 89)	^a^11 (9–13) (*n =* 48)	0.2
APACHE II score	^a^25 (20–30)	^a^24 (18–29)	^a^26 (24–32)	0.004
Plasma 5-HIAA (ng/ml)	^a^9.4 (5.5–24.3)	^a^8.6 (5–16.5)	^a^14.6 (6.9–49.7)	<0.001

*Characteristics of all (n = 157), alive (n = 105), and death (n = 52) during 90 days of follow-up are shown. Between alive and death groups, Fisher's exact tests were used for sex and shock distribution difference analyses, and Mann–Whitney U-tests were used for age, SOFA score, APACHE II score, and plasma 5-HIAA level difference analyses*.

a *Median (interquartile range) are shown*.

**Table 2:** Differences in mortality rate based on clinical variables evaluated by cross-sectional analysis.

	**Univariate analysis**		**Multivariate analysis**	
**Variables (*n =* 157)**	**^**a**^OR (95% ^**b**^CI)**	***p***	**^**a**^OR (95% ^**b**^CI)**	***p***
Sex (female)	0.9 (0.4–1.7)	0.7	–	–
Age	1.03 (1.01–1.06)	0.005	1.02 (0.9–1.04)	0.1
Shock	4.1 (1.7–10)	0.002	3.7 (1.4–9.5)	0.008
APACHE-II score	1.1 (1.02–1.1)	0.005	1.03 (0.9–1.1)	0.3
Plasma 5-HIAA (ng/ml)	1.03 (1.01–1.04)	<0.001	1.02 (1.007–1.04)	0.005

*Binary logistic regression model analyses of the mortality rate during 90 days of follow-up are shown. Variables with significance (p < 0.05) in the univariate analysis were used for multivariate analysis*.

a *OR, odds ratio*;

b *CI, confidential interval range*.

**Table 3:** Mortality rate difference in clinical variables, by longitudinal analysis.

	**Univariate**		**Multivariate**	
**Variables (*n =* 157)**	**^**a**^HR (95% ^**b**^CI)**	**p**	**^**c**^aHR (95% CI)**	***p***
Sex (female)	0.9 (0.5–1.6)	0.8	–	–
Age	1.03 (1.01–1.05)	0.004	1.01 (0.9–1.03)	0.2
Shock	3.3 (1.5–7.3)	0.003	2.7 (1.2–6.0)	0.02
APACHE-II score	1.06 (1.02–1.09)	0.004	1.02 (0.9–1.1)	0.3
Plasma 5-HIAA (ng/ml)	1.01 (1.007–1.02)	<0.001	1.01 (1.003–1.02)	0.005

*Cox hazard model analyses of the mortality rate during 90 days of follow-up are shown. Variables with significance (p < 0.05) in the univariate analysis were used for multivariate analysis*.

a *HR, hazard ratio*;

b *CI, confidential interval range*;

c *aHR, adjusted hazard ratio*.

## Discussion

The pathogenesis of ARDS consists of endothelial-epithelial injury, a dysregulated inflammatory response, and fibrosis. To address its inherent heterogeneity, several studies have focused on categorizing the disease by phenotyping based on clinical characteristics and associated blood biomarkers (2, 8). Although the etiologies of ARDS are diverse and complex, supportive treatments are provided, and the clinical effects of these interventions are often limited. In this study, we revealed that plasma 5-HIAA levels were significantly higher in ARDS cases with shock compared to ARDS cases without shock and its was positively correlated with SOFA score and APACHE-II. Such a strong association between plasma 5-HIAA levels and ARDS disease severity was also identified in the longitudinal mortality rate analysis.

An ideal disease biomarker should have a clear correlation with the disease's pathophysiological factors, and must provide specific contexts such as early diagnosis and guide the successful development of novel therapeutic strategies. However, although numerous studies have been conducted, there are no targeted therapies to treat ARDS. There exists a critical gap between biomarker discovery and translation to clinical use. The clinical and biological heterogeneity of ARDS is a pivotal barrier in identifying effective treatments. However, the development of rapid point-of-care (POC) tests remains a priority. Introducing novel POC tests for biomarkers in blood or other body fluids could lead to potentially significant clinical trials for biomarker-guided, cell-specific therapies (e.g., epithelial targeted therapies or endothelial barrier permeability modifiers) (9).

Recently, ARDS biomarkers have been categorized based on their possible pathogenesis and pathways, and have mostly been assessed as diagnostic and prognostic biomarkers. Candidate diagnostic biomarkers that have been well studied include the following: angiopoietin 2 (Ang-2), high mobility group box nuclear protein 1, interleukin 1 beta, interleukin 1 receptor antagonist, interleukin 6, interleukin 8, macrophage inflammatory protein-1a, extracellular nicotinamide phosphoribosyltransferase (eNAMPT), soluble receptor for advanced glycation end products (sRAGE), vascular endothelial growth factor, selectins, and surfactants (8, 10). Furthermore, researchers have thoroughly investigated candidate prognostic biomarkers, including Ang-2, eNAMPT, sRAGE, protein C, and soluble intercellular adhesion molecule-1 (8, 10). To pursue breakthrough therapeutic strategies, the role of microRNAs (miRNAs) as biomarkers or pharmacologic targets has been increasingly investigated (11). In addition, contrary to conventional chemical detection methods for biomarker candidates, recently, a new method, metabolomics, has been applied, allowing for the simultaneous detection of a large set of metabolites from a single sample (12). However, these markers are still far from successfully transitioning to clinical therapeutic development.

In this study, we adopted the coupled hypothesis from our previous study (6), focusing on the serotonin-ROCK pathway approach for the regulation of vascular permeability in ARDS. Several studies have revealed that ROCK inhibition leads to the attenuation of endothelial vascular hyperpermeability in lung injury (13, 14). Several studies have focused on the analysis of the involvement of serotonin in the pathology of ARDS and targeted serotonin as a therapeutic intervention and have shown that serotonin antagonism improves the clinical efficacy of respiratory failure in animal models and human cases. Serum serotonin levels may be associated with pulmonary hypertension in patients with septic ARDS (15). Serotonin receptor blockage contributes to favorable outcomes in a porcine ARDS model (16). One study indicated that platelet entrapment in the lungs and 5-HT release are in part responsible for early respiratory failure. 5-HT inhibition contributes to a favorable outcome of acute respiratory failure (17). These studies indicate the feasibility of the involvement of serotonin in the pathology of ARDS and targeted serotonin as a therapeutic intervention. In addition, from various perspectives, ARDS research has been drawn into focus by the SARS-CoV-2/COVID-19 pandemic and has come to be conducted based on the recent analysis of severe cases of COVID-19; thus, several papers focusing on the action of selective serotonin reuptake inhibitors (SSRIs) have been published. Fluvoxamine [SSRI and a sigma-1 receptor (S1R) agonist] prevented clinical deterioration of symptomatic COVID-19 in a small placebo-controlled randomized trial in the United States. The authors mentioned that the rationale for administering fluvoxamine is its agonistic effect in attenuating the damaging effects of the inflammatory response (18). A larger randomized placebo-controlled study conducted in Brazil found that patients administered fluvoxamine had a lower risk of hospitalization in a COVID-19 emergency setting or transfer to a tertiary hospital due to COVID-19 deterioration (19). Further, a large observational study conducted in France showed that the use of antidepressants (SSRIs) was significantly and substantially associated with a reduced risk of intubation or death (20). The mechanism underlying the role of SSRIs in these results remains uncertain. However, several hypotheses were discussed in these reports, such as its anti-inflammatory action, antiplatelet activity, and antiviral effects on SARS-CoV-2. Serum serotonin levels were increased in COVID-19 cases, including COVID-19-related ARDS. They focused on platelet hyperreactivity in the pathogenesis of COVID-19. However, COVID-19 non-related ARDS is inconclusive because of the small number and heterogeneity of patients for assessment (21). In addition, one review mentioned a potential benefit of SSRIs in the treatment and prevention of inflammatory lung diseases (e.g., COVID-19, ARDS, COPD, and pneumonia) (22), since SSRIs have anti-inflammatory properties. Serotonin transporter inhibitors and ROCK inhibitors have already been approved and applied for treatment, including certain diseases, depression worldwide, and vasospasm of subarachnoid hemorrhage complications in Japan and China, respectively. As mentioned above, ROCK inhibitors have been applied to animal models of lung injury. However, from the viewpoint of clinical application to humans, approved drugs are limited to Japan and China. As for serotonin antagonism, SSRIs are strongly recognized as drugs in the central nervous system, and the function of peripheral serotonin has been attracting attention, but there are few demonstrative cases in animal experiments targeting lung disorders. For these reasons, the application has not yet progressed to clinical applications. However, as mentioned above, there is a growing body of evidence in COVID-19 clinical studies to promote the application of SSRIs to human patients.

Serotonin storage is abundant in platelets, and activation of platelets is induced by inflammation, and the release of serotonin from platelets increases (23). In addition, since it is stored mostly in the enterochromaffin cells of the intestinal tract (24), the release of serotonin is a possible event due to intestinal barrier dysfunction during septic shock (25, 26). If these two factors are proposed as clinical evaluation indicators in the future, they may become more sensitive to the indicators of the combination of plasma 5-HIAA, platelet activation, and intestinal barrier dysfunction. Clinical indicators that may reflect vascular permeability may be the doses of vasopressors and fluid balance, so attempts to quantify these two indicators and combine the evaluation with plasma 5-HIAA might be more accurate in the evaluation in the future.

In this study, plasma 5-HIAA levels were measured using HP liquid chromatography. However, it is technically possible to perform mass analysis (TOF-MAS) as well. Recently, since more hospital laboratories are introducing TOF-MAS for bacterial identification, measurement of plasma 5-HIAA levels by TOF-MAS, with more rapid time would be available.

Our study has several limitations: First, the involvement of 5-HT-ROCK signaling is highly assumed in the clinical observations of these results in our study; however, the complete mechanism of 5-HT or ROCK-associated vascular permeability regulation could not be evaluated using a molecular basis approach. In our previous study, we demonstrated that plasma 5-HIAA levels can be a predominant biomarker of septic shock severity and a novel role of 5-HT in vascular permeability via the ROCK activation pathway. Given that we showed partial elucidation of the involvement of serotonin/ROCK in the regulation of *in vitro* experiments in our previous study (6), additional work should consider the feasibility of clinical trials using these inhibitors to examine the effect on the regulation of vascular permeability in the lung of ARDS. Second, since this data set provided only ARDS patients, comparative analyses including healthy controls or non-ARDS ICU patients were not available. Third, serotonin levels were not directly measured in the present study. Another study in which plasma serotonin and 5HIAA were measured simultaneously in septic shock subjects revealed that more severe cases showed low-serotonin and high-5-HIAA results over time (27). This trend may be because activated serotonin is immediately metabolized to 5-HIAA, which matches with our hypotheses and results. Fourth, the changes in plasma 5-HIAA levels over time were not monitored because of limited data sources. The trend in the time course of plasma 5-HIAA levels would have revealed a more precise understanding of the association between serotonin and vascular leakage in the treatment clinical course.

As a general perception of the pathophysiology of ARDS, there is considerable crosstalk in its pathogenesis (2, 8), and a variety of causes of ARDS also make it difficult to discuss its pathogenesis in a simple manner. We think that the results of our study do not necessarily reflect the whole and accurate phenomenon of ARDS, possibly reflecting one aspect among many. We believe that the same composition of difficulties in showing a positive result study of ARDS in several clinical trials. We would like to emphasize that the biomarkers (plasma 5-HIAA) demonstrated in this study could be used as prognostic factors, in addition to the existing therapeutic drug candidates (SSRIs or ROCK inhibitors) that can directly regulate vascular hyperpermeability.

In this study, we demonstrated the possibility of using plasma 5-HIAA as a prognostic biomarker of ARDS severity. Plasma 5-HIAA (a serotonin metabolite) could be an ideal and unique target to allow the successful transition of a novel clinical treatment targeting the serotonin-ROCK pathway through biomarker development. Furthermore, comprehensive *in vitro* experiments and clinical data assessment are required to reveal the detailed involvement of the serotonin and ROCK pathways in ARDS pathogenesis.

## Data Availability Statement

The original contributions presented in the study are included in the article/supplementary material, further inquiries can be directed to the corresponding author.

## Ethics Statement

The studies involving human participants were reviewed and approved by the Institutional Review Board of Nagasaki University Hospital (approval number 16072514). The NHLBI Research Materials Distribution Agreement (RMDA) was concluded between Nagasaki University Hospital and NHLBI. Written informed consent for participation was not required for this study in accordance with the national legislation and the institutional requirements.

## Author Contributions

TT designed the study and wrote and edited the original draft of the manuscript. MM performed statistical analyses. TT and MM acquired the data set. MT reviewed the study design and edited the manuscript. KI supervised the study and edited the manuscript. All authors approved the study design and manuscript.

## Funding

This study was supported by grants from JSPS KAKENHI, grant 2016–2019 [16K09544].

## Conflict of Interest

The authors declare that the research was conducted in the absence of any commercial or financial relationships that could be construed as a potential conflict of interest.

## Publisher's Note

All claims expressed in this article are solely those of the authors and do not necessarily represent those of their affiliated organizations, or those of the publisher, the editors and the reviewers. Any product that may be evaluated in this article, or claim that may be made by its manufacturer, is not guaranteed or endorsed by the publisher.

## Abbreviations

ARDS: acute respiratory distress syndrome
5-HIAA: 5-hydroxyindoleacetic acid
ROCK: RhoA/Rho-associated protein kinase
5-HT: 5-hydroxytryptamine
SOFA: Sequential Organ Failure Assessment
APACHE: Acute Physiology and Chronic Health Evaluation
SSRIs: selective serotonin reuptake inhibitors
POC: point-of-care
Ang-2: angiopoietin 2
eNAMPT: extracellular nicotinamide phosphoribosyltransferase
sRAGE: soluble receptor for advanced glycation endproducts
miRNAs: microRNAs.
